# Characterization of the D8P1C1 Anti-ADAM17 Inhibitory Monoclonal Antibody and Generation of Its Bispecific T-Cell Engager Derivative

**DOI:** 10.3390/ijms27072936

**Published:** 2026-03-24

**Authors:** Nayanendu Saha, Sang Gyu Lee, Elisa de Stanchina, Rachelle P. Mendoza, Darren R. Veach, Dimitar B. Nikolov

**Affiliations:** 1 Structural Biology Program, Memorial Sloan Kettering Cancer Center, New York, NY 10065, USA; sahan@mskcc.org; 2 Department of Radiology, Memorial Sloan Kettering Cancer Center, New York, NY 10065, USA; 3 Antitumor Assessment Facility, Memorial Sloan Kettering Cancer Center, New York, NY 10065, USA; 4 Department of Pathology and Cell Biology, Columbia University Irving Medical Center, New York, NY 10032, USA

**Keywords:** ADAM17, bispecific T-cell engager, high-grade serous ovarian cancer, EGFR signaling

## Abstract

EGFR signaling, which requires ligand shedding by ADAM proteases, drives the progression of a variety of cancers, including breast, ovarian and lung. We previously reported the generation and characterization of a fully human, affinity-matured anti-ADAM17 monoclonal antibody, D8P1C1, which inhibits both the proliferation of an array of cancer cell lines in vitro as well as breast cancer growth in a mouse xenograft model. Here, we show that the mAb inhibits the shedding of EGFR ligands and EGFR phosphorylation in cancer cell lines, thus explaining its anti-tumor effects. In a xenograft model with a high-grade serous ovarian cancer (HGSOC) cell line, D8P1C1 showed only modest therapeutic effect, without any discernible toxicity. These results suggest that ovarian cancers are less susceptible than breast cancers to therapeutic targeting of ADAM17- or EGFR-dependent signaling. Radioimmuno PET imaging with ^89^Zr-DFO-D8P1C1 confirmed tumoral accumulation of the mAb in high-grade and non-high-grade serous ovarian tumor xenografts. Furthermore, we report the generation and preliminary characterization of a bispecific T cell engager derivative of D8P1C1 with improved anti-tumor efficacy in vitro.

## 1. Introduction

The human epidermal growth factor receptor (EGFR) family of tyrosine kinases is implicated in the development and proliferation of many human cancers, notably breast, lung, ovarian, prostate and colon [1]. Activation of EGFR signaling requires ligand shedding by ADAM17, and it has been documented that there is a strong correlation between high EGFR activation and high ADAM17 expression levels in cancer progression [2,3,4,5]. For instance, ADAM17-dependent EGFR ligand shedding was shown to be an important proliferative signal in triple-negative breast cancer (TNBC) [6]. Likewise, ADAM17 is upregulated in ovarian tumors and in turn activates EGFR signaling [7]. Because of the pivotal role of ADAM17 in the proteolytic shedding of cell-surface attached EGFR ligands, which triggers oncogenic signaling, targeted inhibition of ADAM17 is an efficient means of therapeutic intervention, particularly in solid tumors [8].

ADAMs are transmembrane proteins with an N-terminal pro-domain, followed by metalloprotease (MP), disintegrin (D), cysteine-rich (C), transmembrane and cytoplasmic domains [9]. ADAMs do not exhibit any typical substrate cleavage signature; rather, the specificity is inherent to interactions of the D + C domain region of the proteases with the substrates [10,11,12,13]. In addition, the ADAM metalloprotease domains display a high degree of sequence conservation, and, consequently, small-molecule inhibitors targeting the proteinase active site proved inefficient in clinical trials due to a lack of specificity [14].

To target ADAM17, we previously reported a fully human, affinity-matured, inhibitory, monoclonal antibody (mAb), D8P1C1, which recognizes ADAM17 expressed on cancer cells and inhibits the proliferation of a wide array of tumor cells in cell-based assays [15]. In xenograft assays with the TNBC cell line MDA-MB-231 and a non-high-grade serous ovarian cancer line, D8P1C1 caused 78% and 45% tumor growth inhibition, respectively, with no discernible toxicity effects [15]. Here, we undertake further investigation of the efficacy of the D8P1C1 mAb in inhibiting shedding of cell-bound EGFR ligands and EGFR phosphorylation in a variety of cancer cells. In a xenograft study with a high-grade serous ovarian cancer (HGSOC) cell line, the mAb exhibited only marginal efficacy without any discernible toxicity. Consistent with its preferential tumor cell binding in vitro, positron emission tomography/computed tomography (PET/CT) imaging and biodistribution analysis in mice bearing HGSOC and non-HGSOC xenografts confirmed tumoral accumulation of the D8P1C1 mAb. Finally, using this mAb as a platform, we generated and evaluated in vitro a bispecific T-cell engager antibody (BiTE) [16], which displays an improved therapeutic efficacy towards breast and ovarian cancer cells in the presence of peripheral blood mononuclear cells (PBMC). Given the unmet need for targeted therapies to treat solid cancers, including breast (TNBC in particular) [17] and ovarian (epithelial in particular) [18], ADAM17-specific BiTEs could provide a novel avenue to replace or supplement existing treatment regimens.

## 2. Results

### 2.1. Purification of the D8P1C1 mAb

We previously reported the generation of the D8P1C1 mAb [15]. D8P1C1 was expressed in Expi 293 cells and was purified to ≥99% homogeneity using protein A Sepharose, followed by SEC-HPLC (column TSKgel G3000SWxl, Tosoh Bioscience, King of Prussia, PA, USA). On SDS-PAGE, under non-reducing conditions, D8P1C1 migrated at 150 kDa. Under reducing conditions, the heavy and light chains migrated at 50 and 25 kDa respectively (Figure S1). In biolayer interferometry (BLI) assays, the mAb D8P1C1 bound to the ADAM17 extracellular domains region (ADAM17-ECD) with a KD of 180 pM (Figure S2), which is similar to another anti-ADAM17 mAb MED13622 (MedImmune, Gaithersburg, MD, USA) [19]. However, D8P1C1 was more potent than MED13622 in inhibiting the proliferation of the TNBC cell line MDA-MB-231 in vitro (Figure S3).

### 2.2. The D8P1C1 mAb Blocks EGFR Ligand Shedding from Cancer Cells with High Efficiency, While Its Effect on the Cleavage of Other ADAM17 Substrates Varies

Cancer cell lines that are known to express EGFR ligands, such as EGF, TGFα, and AREG, were selected, and sandwich ELISA-based shedding assays were performed to gauge the effect of D8P1C1 mAb on the release of the cell-tethered ligands into the culture supernatant. It has been documented that the ADAM17-mediated shedding of TGFα and EGF governs disease processes, including cancer [20] and inflammation [21]. Post-shedding, soluble forms of EGFR ligands bind and activate EGFR in an autocrine or paracrine manner. This leads to the onset of downstream signaling pathways triggering events, such as migration and cell invasion, that culminate in metastasis. For instance, ADAM17-dependent shedding of TGFα and EGF promotes invasion and cell migration of TNBC (MDA-MB231) cells [22,23]. Interestingly, in ovarian cancer cells, TGFα and EGF can stimulate or repress cell growth and DNA synthesis depending on the cell line. In the HGSOC OVCAR-3 cells, TGFα and EGF promote DNA synthesis and cell growth, while exhibiting opposite effects in Caov-3 cells. EGF is also known to stimulate gonadotropin-releasing hormone synthesis and facilitate invasiveness in OVCAR-3, Caov-3, and SKOV-3 cells [24]. Shedding of AREG by ADAM17 promotes ovarian cancer cell proliferation, metastasis, stemness, and therapy resistance. AREG can also modulate the tumor immune microenvironment by tuning the expression of immune evasion genes, such as CXCL8, CXCL1, and CXCLq [25].

In addition to EGFR ligands, we monitored the effect of the anti-ADAM17 mAb on the shedding of other known ADAM substrates, such as TNFα [26], CX3CL1 [27] and Notch [28], which also play central roles in cancer progression. TNFα acts as a pro-tumorigenic factor and abets intramural invasion of TNBC cells [26], while the chemokine CX3CL1 [27], which can exist in membrane-bound or soluble form, interacts with its receptor CX3CR1 and mediates progression of non-small cell lung cancer [28].

**Figure 1:**
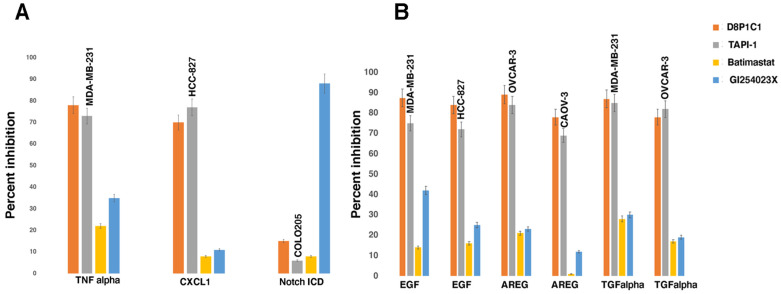
D8P1C1 (20 µg/mL) inhibits the shedding of EGFR ligands and other ADAM17 substrates on the cell surface. Sandwich ELISA was employed to quantitate the shedding (or cleavage) of ADAM17 substrates (**A**) and EGFR ligands (**B**). For Notch, we measured the release of the Notch intracellular domain or NICD1 in the cell lysates. Percent inhibition of shedding by the antagonists was calculated by measuring the decrease in shedding observed in comparison to the untreated wells. The cell lines used in this assay were MDA-MB-231, HCC-827, COLO205, OVCAR-3, and Caov-3. The small-molecule inhibitors include TAPI-1 (ADAM17 inhibitor, but can also inhibit other ADAMs), Batimastat (broad-spectrum MMP inhibitor), and GI254023X (ADAM10-specific inhibitor). The data represent the mean of quadruplicate determinations (n = 4), and the bars on the graph show the effect of administration of antagonists on the different cancer cells (as indicated above the bars) relative to the control (untreated cells), mean ± SEM.

The shedding assays (Figure 1A,B) show that D8P1C1 mAb inhibits ectodomain shedding of EGFR ligands (EGF, TGFα, and AREG) with similar efficiency (78–87% inhibition) in all cancer cell lines that were examined. Regarding the other ADAM substrates, D8P1C1 inhibited TNFα and CX3CL1 quite strongly, 78% and 70% respectively, consistent with ADAM17 being the preferred sheddase. The inhibition was down to 15% for Notch1, as evaluated from the release of Notch intracellular domain 1 (NICD1), which corroborates our earlier studies that ADAM10 is the principal sheddase in the Notch pathway [13] (*p* < 0.001 for all of the above).

TAPI-1 [29], a small-molecule inhibitor that binds to the ADAM17 protease active site, showed a pronounced effect under all conditions, while the anti-ADAM10 small-molecule inhibitor GI254023X [30] was only effective in impeding Notch cleavage (*p* < 0.001). Batimastat, a broad-spectrum matrix metalloproteinase (MMP) inhibitor, had minimal effect on the shedding of the EGFR ligands and the other ADAM17 substrates. The isotype control IgG1 had no effect on the shedding of EGFR ligands or ADAM substrates (Figure S4).

### 2.3. D8P1C1 Inhibits EGFR Phosphorylation in MDA-MB-231, HCC827, OVCAR-3, and SKOV-3 Cells

**Figure 2:**
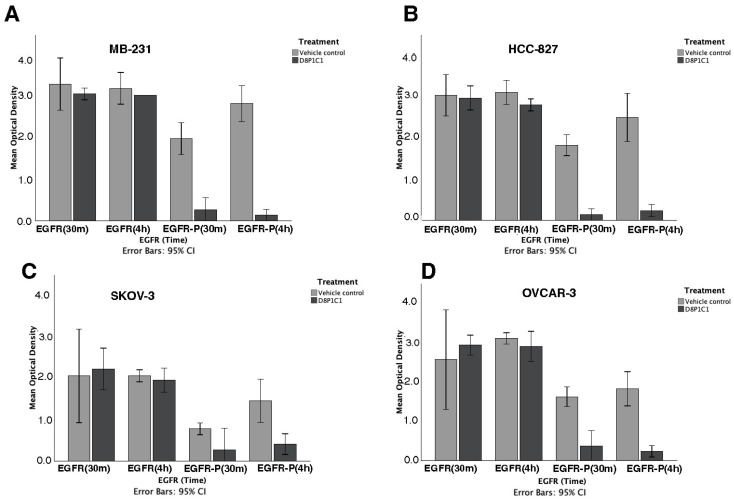
D8P1C1 inhibits EGFR/erbB phosphorylation in cancer cells in vitro. Sandwich ELISA was used to measure the levels of total EGFR and phosphorylated EGFR (EGFR-P) in MDA-MB-231 (**A**), HCC827 (**B**), SKOV-3 (**C**), and OVCAR-3 (**D**) cells upon treatment with 10 µg/mL of D8P1C1. The data represent the mean of triplicate experiments (n = 3), and the bar plots show the effect of treatment with D8P1C1 relative to untreated control, mean ± 95% CI. Comparison of EGFR levels between treated and untreated groups was performed using the independent *t* test. Total EGFR levels after 4 h of incubation did not significantly differ between the two groups in MDA-MB-231 (*p* = 0.132), OVCAR-3 (*p* = 0.101) and SKOV-3 (*p* = 0.251). However, in HCC827, the small but statistically significant difference could be due to a decrease in the total number of viable cells (*p* = 0.016). On the other hand, the mAb-treated group showed a significant decrease in the EGFR-P levels in all cell types, as compared to the untreated control, *p* < 0.001.

We estimated the levels of total EGFR and phosphorylated EGFR (EGFR-P), with or without D8P1C1 mAb treatment (after 30 min and after 4 h of incubation, using sandwich ELISA kits, PathScan Cell Signaling Technologies, Danvers, MA, USA [31]), in MDA-MB-231, HCC-827, OVCAR-3 and SKOV-3 cells that are known to express EGFR. After 4h of incubation with the mAb, we observed 95% inhibition of phosphorylation in the TNBC cell line MDA-MB-231 and 91% inhibition in the NSCLC line HCC-827. The inhibitory effect was slightly lower in the ovarian cancer cells OVCAR-3 (87% inhibition) and SKOV-3 (71% inhibition), as shown in Figure 2 (*p* < 0.001). These results correlated well with the results from the EGFR ligand shedding assays (Figure 1), as shedding of EGFR ligands is a prerequisite for the activation of EGFR signaling. Indeed, cleaved EGFR ligands bind to the extracellular domain of the receptors, triggering a conformational change leading to EGFR dimerization and autophosphorylation. These events are central to activating downstream signaling associated with cell growth and proliferation. Thus, it is likely that the D8P1C1 mAb affects EGFR phosphorylation by drastically depleting the levels of soluble/shedded EGFR ligands (see Figure 1 above).

### 2.4. Xenograft Assay to Estimate the Anti-Tumor Potential of D8P1C1 in OVCAR-3 HGSOC Cells

**Figure 3:**
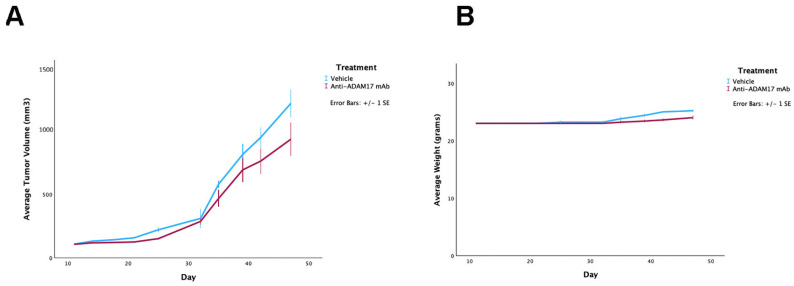
Xenograft model using OVCAR-3 ovarian cancer cells implanted into female NSG mice. (**A**) The anti-ADAM17 mAb D8P1C1 causes 25.4% percent tumor growth inhibition at a dose of 40 mg/kg (although this was not statistically significant, *p* = 0.07), administered bi-weekly for 4 weeks; 6–8 weeks old NSG mice (n = 5, number of mice per group) were used. Graphs show mean ± SEM. (**B**) Body weight monitoring throughout the treatment period. The control treatment was PBS, which is commonly used in OVCAR-3 xenografts [33,34].

Previously, we showed that the D8P1C1 mAb moderately inhibited the non-HGSOC cells, SKOV-3, in vivo [15]. It was pertinent, therefore, to evaluate its anti-tumor potency also, using HGSOC cells such as OVCAR-3. At a dose of 40 mg/kg, D8P1C1 treatment only marginally affected tumor size, decreasing tumor volume by an average of 25.4% (on day 47 after tumor cell implantation), with no indication of mAb-induced toxicity in the mice (e.g., no diarrhea or decrease in body weight), even though the mAb recognizes equally well both mouse and human ADAM17 (Figure 3). Lack of toxicity, even at a high dose, is consistent with D8P1C1 targeting activated ADAM17 in tumors [15]. It can be speculated that the absence of a more pronounced inhibition of tumor growth with the OVCAR-3 cells (as compared to the observed 45% tumor growth inhibition with SKOV-3 cells [15].) could be attributed, in part, to the prevalence of mutations in TP53, a key player in the progression of HGSOC, such as OVCAR-3 [32].

### 2.5. Radiolabeling, PET/CT Imaging and Biodistribution Studies to Investigate the In Vivo Pharmacokinetics and Distribution of D8P1C1 In Vivo

**Figure 4:**
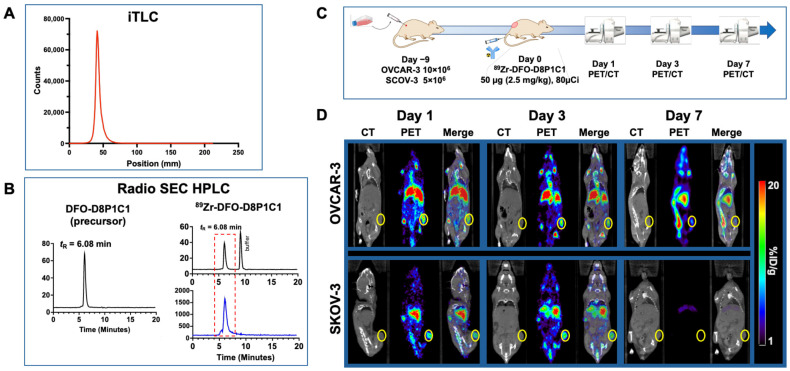
Experimental scheme and representative images of PET/CT of ^89^Zr-DFO-D8P1C1 antibodies in OVCAR-3 and SKOV-3 xenografts model. (**A**) iTLC chromatogram of ^89^Zr-DFO-D8P1C1. Zr-89 radiometallation of DFO-conjugated D8P1C1 is efficient (>99% radiochemical yield). (**B**) Radio-SEC-HPLC chromatograms of DFO-conjugated D8P1C1 (left) and radiolabeled ^89^Zr-DFO-D8P1C1 (right). DFO conjugation and radiolabeling have minimal impact on SEC retention time and minimal aggregates. (**C**) Experimental scheme. NSG mice were inoculated with 10 × 10^6^ OVCAR-3 or 5 × 10^6^ SKOV-3 cells on the right flank of the mice (n = 3, number of mice for each ovarian cancer line, for each time point). Nine days after inoculation, 50 µg ^89^Zr-DFO-D8P1C1 (3 MBq, 80µCi) was administered intravenously, and serial PET/CT imaging was performed. (**D**) Representative coronal PET/CT images at 1, 3 and 7 days post-injection. Tumors were clearly delineated with ^89^Zr-DFO-D8P1C1 PET/CT imaging in both ovarian cancer models, with longer tumor retention in OVCAR-3.

Biological and receptor-based processes can be probed quantitatively using radiolabeled PET probes. Zirconium-89 has become the PET isotope of choice to track mAb pharmacokinetics in vivo because its 3.3-day half-life matches the clearance kinetics of most biological molecules [35]. Direct chemical conjugation of the Zr-89 chelator DFO does not appreciably change the physicochemical characteristics at low chelator-to-mAb ratios, and Zr-89 radiolabeling technology is robust and well-established. The DFO conjugated (DFO-D8P1C1 mAb) and radiolabeled products were purified by gel filtration and characterized by radio-SEC HPLC and iTLC (Figure 4A,B). Nearly quantitative incorporation of ^89^Zr (>99% radiochemical yield) was obtained at a reasonable specific activity (A_S_ = 59 MBq/mg, 1.6 mCi/mg) required for PET imaging. The DFO conjugation and radiolabeling had no appreciable impact on SEC-HPLC retention time compared to the reference standard, nor was there evidence of radiolabeled fragments, nor evidence of significant aggregation. In vivo evaluation was performed according to the workflow described in Figure 4C. Subcutaneous (s.c.) xenografts of OVCAR-3 or SKOV-3 were established in NSG mice, and ^89^Zr-DFO-D8P1C1 was administered in the tail vein in a bolus injection. Serial micro-PET/CT imaging of tumor-bearing mice was performed at 24, 72 and 168 h post-injection. Immediately after the last imaging time point, ex vivo biodistribution was performed. Mice were euthanized by CO_2_ (gas) asphyxiation and tissues, including the tumor, were removed. Metabolically released free Zr-89 metal accumulated in the bone/tibia; this is unlikely due to free Zr-89 in the injectate, since it would be eliminated during the purification. The PET/CT data (Figure 4D) showed that the D8P1C1 mAb accumulated in SKOV-3 and OVCAR-3 tumors, persisted in the blood, gut, and liver, and cleared from most other tissues. Overall, this data indicates that the highest contrast ratios, and thus most favorable imaging time post radiotracer, are observed three days after radiotracer administration. ^89^Zr-DFO-D8P1C1 was durably retained on the tumor in both models to day 3, but the tracer appeared to undergo systemic clearance faster in the SKOV-3 model by day 7.

**Figure 5:**
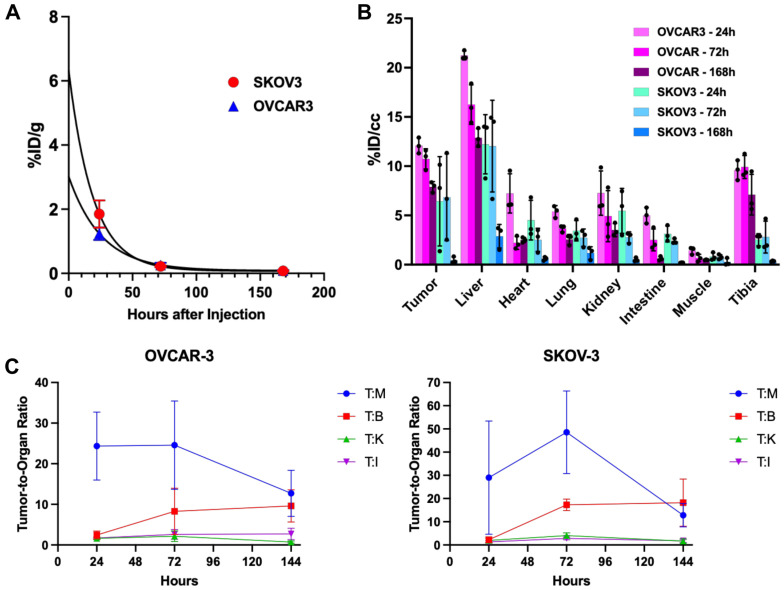
Pharmacokinetics and in vivo distribution of Zr-89 labeled D8P1C1. (**A**) Blood time-activity curves of ^89^Zr-DFO-D8P1C1 in OVCAR-3 and SKOV-3 xenografted mice. Serial blood samples were counted and represented in percent decay-corrected injected dose per gram (%ID/g; n = 5). Blood half-life of D8P1C1 was 17.8 ± 2.5 and 13.2 ± 1.7 h in OVCAR-3- and SKOV-3-bearing mice, respectively. (**B**) Longitudinal PET ROI-analysis-based organ biodistribution. ^89^Zr-DFO-D8P1C1 uptake in tumor and critical organs was quantified using PET/CT images (n = 3, number of mice per group, mean ± SD). (**C**) Tumor-to-organ ratios. T:M = tumor to muscle (a measure of general background), B = blood/heart, K = kidney, and I = intestine/gut in OVCAR-3 (**left**) and SKOV-3 (**right**).

Region of interest (ROI) analysis of the longitudinal PET/CT data (Figure 5B,C and Table 1) indicated that tumor uptakes of D8P1C1 at 72 h are 10.74 ± 1.03%ID/cc for OVCAR-3 and 6.85 ± 4.41%ID/cc for SKOV-3. The differential PET uptake between SKOV-3 and OVCAR-3 was likely a result of the slower growth of SKOV-3 at the time of PET imaging (<50 vs. 200 mm^3^). Overall, the longitudinal biodistribution data indicate that the highest contrast ratios, and thus most favorable imaging time post radiotracer, were observed three days after radiotracer administration. ^89^Zr-DFO-D8P1C1 was durably retained on the tumor in both models to day 3, but the tracer appeared to undergo systemic clearance faster in the SKOV-3 model by day 7, presumably due to a difference in tumor volume and sink effect, and possibly due to differential epitope dynamics. Previously, we reported PET imaging and biodistribution studies with another anti-ADAM17 mAb, C12, that targets the D + C domains [36]. Comparative profiles of D8P1C1 and C12 mAbs indicated that the D8P1C1 mAb is superior to C12 in targeting tumors in both the OVCAR-3 and SKOV-3 xenografts.

### 2.6. Generation and Characterization of D8P1C1 BiTE

**Figure 6:**
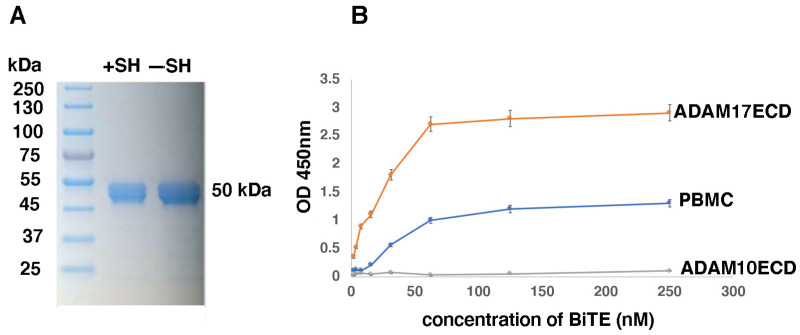
SDS-PAGE (**A**) and an ELISA-based binding assay (**B**) with the D8P1C1-BiTE. (**A**) On SDS-PAGE, under reducing (+SH) and non-reducing (−SH) conditions, the BiTE migrates at 50kDa. The BiTE migrates as a single peak corresponding to 50 kDa on SD-200 (SEC) and was purified to 98% homogeneity. The D8P1C1-BiTE specifically binds to immobilized ADAM17-ECD and not to ADAM10-ECD (ADAM10 is closely related to ADAM17), thus retaining its specificity for ADAM17. Likewise, it recognizes human peripheral blood mononuclear cells (PBMC) immobilized on ELISA plates. The Fc-tagged version of the BiTE, just after protein A Sepharose elution, was used for the ELISA assay. The bound Fc-tagged BiTE was detected by goat anti-human Fc conjugated to HRP (Jackson Immunoresearch, West Grove, PA, USA). Color was developed using the TMB substrate kit (Thermo Fisher Scientific, Waltham, MA, USA). The data, the mean of triplicate determinations (n = 3), was recorded at 450 nm.

Since at a high dose (40 mg/kg) the D8P1C1 mAb did not cause toxicity in mice, it is logical to anticipate that a BiTE derivative is also likely to be devoid of non-specificity and toxicity issues. To generate the BiTE derivative of D8P1C1, we fused the scFv’s of D8P1C1 [15] and an anti-CD3 mAb [16,37] (Figure S5) and expressed and purified the product. This BiTE is expected to bind and inhibit ADAM17 on tumors, while recruiting cytotoxic T cells, thus engaging the immune system to fight the disease. This improves the efficacy of cancer immunotherapies by enhancing T cell infiltration into tumor tissues. Size-exclusion chromatography (Figure S6) and SDS-PAGE analysis (Figure 6A) showed that the D8P1C1 BiTE migrates with the expected native molecular weight of 50 kDa. We performed ELISA-based binding studies to confirm that the BiTE retains binding to both ADAM17 and PBMC. Briefly, we coated wells of the ELISA plate (Nunc) with ADAM17-ECD or ADAM10-ECD (at 1 µg/mL). PBMCs (1 × 10^5^ cells/mL) were coated in the presence of 1% paraformaldehyde. The results showed that the BiTE specifically recognizes the ADAM17-ECD and not the ADAM10-ECD construct. Likewise, it is bound to immobilized PBMCs. The D8P1C1 mAb (IgG or Fab) did not bind to PBMC (Figure S7A), indicating that the anti-CD3 scFv of the BiTE mediates the binding of the BiTE to PBMC. Likewise, we did not observe the binding of the BiTE or D8P1C1 (IgG or Fab) to immobilized HL-60 (acute promyelocytic leukemia) cells (Figure S7B). Indeed, the well-characterized [37,38,39] anti-CD3 scFv module that we used is highly specific for the epsilon chain of human CD3, which is only present in the T cells within the PBMC mixture [40]. Thus, the binding specificities for ADAM17 and CD3 were not affected during BiTE construction or expression (Figure 6B). Comparison of anti-tumor efficacies of the BiTE (in the absence of PBMC) and the unmodified D8P1C1 in Alamar blue assays, using the breast cancer cell line MDA-MB-231 (TNBC), ascertained that the inhibitory, anti-tumor activity of the BiTE was not compromised (Figure S3). In biolayer interferometry (BLI) assays, the BiTE bound to ADAM17 ECD with a K_D_ of ~130 pM, which is similar to the parental mAb (Figure S2).

### 2.7. Activation of PBMC (T Cells) by the D8P1C1 BiTE

**Figure 7:**
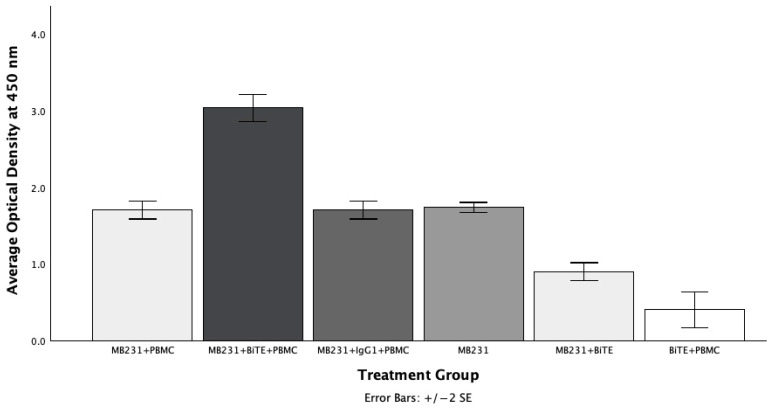
The D8P1C1 BiTE activates the T cells (in the PBMC mixture) in the presence of target cells. Sandwich ELISA was employed to quantitate the release of TNFα in the culture supernatant of MDA-MB-231 cells. Pathscan sandwich ELISA was performed [31]. The data represent the mean of triplicate determinations (n = 3). The addition of the BiTE significantly increased the release of soluble TNFα in the PBMC: target-cell mixture (*p* < 0.001), while the IgG1 control had no effect (*p* > 0.9).

To assess if the D8P1C1 BiTE activates T cells, PBMCs from healthy donors (ATCC) were mixed with MDA-MB-231 cells (target cells) in 96-well tissue culture plates, and the levels of secreted TNFα were measured. The assay was performed as described previously [39]. The target-cells:PBMC ratio was maintained at 5:1. BiTE (1 µg/mL) was added to the MDA-MB-231/PBMC mixture in conditioned media and incubated for 24 h. Then, the culture supernatant was harvested and centrifuged, and the release of TNFα was determined using the sandwich ELISA kit [31]. The results were compared to the release of TNFα from the same MDA-MB-231+PBMC mix but not treated with antibody reagent, as well as to an isotype control, IgG1+MDA-MB-231+PBMC. We also included the following combinations in our assay: MDA-MB-231 cells alone, MDA-MB-231+BiTE, and PBMC+BiTE (no target cells) in our assays. The results (Figure 7) clearly document that the BiTE significantly boosted the release of the cytokine when both MDA-MB-231 and PBMC are present (*p* < 0.001), while the IgG1 control had no effect on cytokine release (*p* > 0.9). It should be noted that the MDA-MB-231 cells alone release endogenous TNFα via proteolysis by ADAM17, and the BiTE partially inhibits this release due to the inhibitory effect of the D8P1C1 arm on ADAM17 (*p* < 0.001). In T cells, in addition to ADAM17, proteases such as ADAM10 and MMP [41,42] that can also cleave TNFα are present, and therefore such an inhibitory effect is not pronounced. Indeed, it has been previously shown that even a complete deletion of ADAM17 does not block the release of TNFα from the surface of activated T cells [43]. Overall, the data suggest that in the presence of target cancer cells (MDA-MB-231), the anti-CD3 arm of the BiTE engages CD3 and activates the T cell population in the PBMC milieu, leading to an increase in cytokine release.

### 2.8. Cell Viability Assays in the Presence of PBMC Document That the D8P1C1-BiTE Is More Potent in Inhibiting Breast and Ovarian Cancer Cell Lines than the Parental D8P1C1 mAb

**Figure 8:**
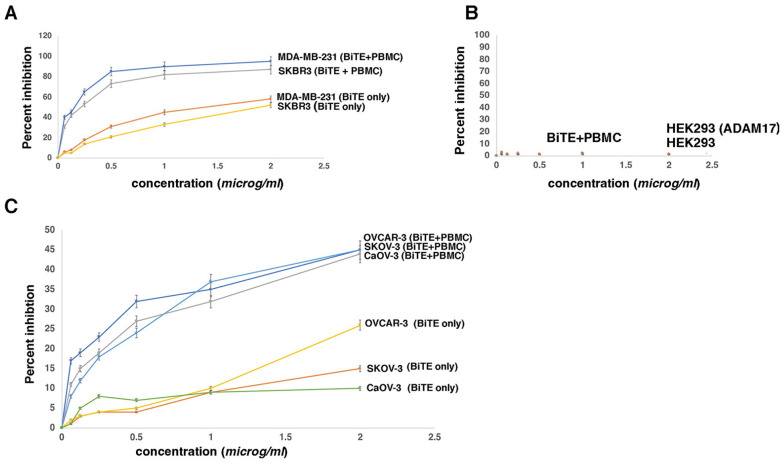
Anti-tumor potency of the D8P1C1-BiTE in the presence and absence of PBMC. An Alamar blue cell viability assay was performed to evaluate the anti-tumor potency of the D8P1C1-BiTE in the presence and absence of PBMC. The cell lines used include breast cancer (MDA-MB-231, SKBR-3) (**A**), as well as serous ovarian (OVCAR-3, SKOV-3, and Caov-3) (**C**) lines. We also performed the cell viability assay using untransfected HEK 293, as well as HEK293 cells transfected with human ADAM17 (wild-type full-length construct) (**B**). The data represent the mean of triplicate determinations and two independent experiments (n = 6). Maximum dispersion was within 10% of the mean value. The X-axis represents BiTE concentration in microg/mL (1 microg/mL is equivalent to ~20 nM for a BiTE).

Next, we determined the anti-tumor potency of the BiTE in the presence of PBMC using breast (MDA-MB-231, SKBR-3), as well as serous ovarian (OVCAR-3, SKOV-3, and Caov-3) cancer lines. Similar to the T cell activation assay, we maintained the target-cells: PBMC ratio at 5:1. The highlights of the Alamar blue cell viability assays [44,45] were as follows: (i) In the presence of PBMC, the BiTE was significantly more potent in breast and ovarian tumor-cell killing, as compared to the BiTE administered alone (*p* < 0.001), with the latter having a similar anti-tumor profile as the parental D8P1C1 mAb (Figure S3). For instance, at 1 µg/mL, the BiTE was two to three-fold more effective in killing breast cancer cells in the presence of PBMC. With the ovarian cancer cells, the increase in potency was between three and five-fold (Figure 8A,C). (ii) In the presence of PBMC, HEK293 cells transfected with an ADAM17 construct were unaffected, suggesting that the BiTE does not target the autoinhibited ADAM17 expressed on HEK293 and non-tumor cells [15] (Figure 8B). (iii) Overall, the BiTE was more effective in killing breast cancer cells (as compared to ovarian cancer cells), possibly due to the increased prevalence of the ADAM17-EGFR signaling axis in driving breast cancer progression. It should be noted that, in general, BiTEs are more potent at doses lower than whole IgGs, and BiTEs have been successfully used for cancer treatment. An important example is non-Hodgkin’s lymphoma, where a BiTE, specific for CD19 on B-cells and CD3 on T cells [46], led to complete tumor regression in patients. Amongst others, BiTE antibodies specific for CD3 and EpCAM [37] or CD3 and EGFR have shown considerable promise in preclinical studies with xenograft models [47].

## 3. Discussion

Here, we characterize in further detail a novel inhibitory anti-ADAM17 mAb, D8P1C1, which we previously reported [15]. Our previous studies highlighted that D8P1C1 recognizes an activated ADAM17 conformation expressed on the surface of cancer cells and inhibits the proliferation of cancer cells in vitro and in vivo [15]. Now, we show that D8P1C1 acts via preventing the shedding of a variety of EGFR ligands and other ADAM17 substrates, and that this inhibition results in a potent decrease in EGFR/erbB phosphorylation in breast, ovarian and lung cancer cell lines. The highly efficient inhibition of EGFR ligand shedding and EGFR phosphorylation in breast cancer cell lines correlates well with the D8P1C1’s anti-tumor effects against these cell lines both in vitro and in vivo [15]. In comparison, while D8P1C1 inhibits EGFR-ligand shedding and EGFR phosphorylation in ovarian cancer cell lines well, it displays only modest anti-tumor effects against these cell lines.

In our previous study with D8P1C1, we performed xenograft studies on mice with the highly aggressive TNBC line (MDA-MB-231) and the epithelial ovarian cancer cell line, SKOV-3, which is of non-HGSOC subtype. Now, we conducted similar xenograft studies with another epithelial ovarian cancer line, OVCAR-3, which is of the HGSOC subtype. Epithelial ovarian cancer is the most common type of ovarian cancer [48]. Treatment for ovarian cancer usually involves a combination of surgery and chemotherapy. Chemotherapeutic resistance is a major cause of mortality [49]. Treatments with poly ADP ribose polymerase inhibitors (PARP) have rekindled some degree of promise in quelling recurrent ovarian cancer [50]. It has been demonstrated that augmented ADAM17 proteolytic activity facilitates receptor activation and tumor survival and is regarded as a potential chemoresistance mechanism [51]. Consequently, ADAM17 inhibition by a small-molecule inhibitor, such as GW280264X, increases the impact of the chemotherapeutic drug cisplatin in treating epithelial ovarian cancer spheroids [52]. In our studies presented here, D8P1C1 impeded the proliferation of OVCAR-3 tumors in vivo only to a modest extent, with no toxicity effect even at a high dose. This is consistent with earlier studies conducted with SKOV-3 cells [15]. Indeed, in our previous studies with SKOV-3 xenografts, we observed 45% tumor growth inhibition at a dose of 60 mg/kg [15], comparable to the in vivo inhibition of OVCAR-3 tumors reported here (25% at 40 mg/kg). HGSOC (OVCAR-3) is marked by extensive genomic instability [50] and TP53 mutations [32], whereas non-HGSOC (SKOV-3) is usually driven by the MUC16 pathway [53]. The exact role of ADAM17 in mediating these distinct pathways is unclear, and our studies suggest that the inhibition of EGFR signaling alone is not sufficient for blocking the proliferation of ovarian cancer cells in vitro or in vivo.

PET imaging and biodistribution studies with the OVCAR-3 and SKOV-3 cells indicate that the Zr-89 radiolabeled D8P1C1 mAb detects the expression of activated ADAM17 in human ovarian tumor xenografts. ^89^Zr-DFO-D8P1C1 durably accumulated in SKOV-3 and OVCAR-3 tumors and had a reasonable rate of blood clearance (t_1/2_ = 13.2 and 17.8 h for OVCAR-3 and SKOV-3-implanted mice respectively, Figure 5A,B). Presumed internalization after specific binding, despite evident metabolic processing of the ^89^Zr-DFO-D8P1C1, justifies that activated ADAM17 is a feasible target for antibody and antibody-based therapeutics in ovarian cancer. Since PET is a quantitative technique, the intratumoral drug concentration can be calculated over time. At a moderate IV tracer dose of 2 mg/kg, D8P1C1 approached 4.0 nM and 2.1 nM in OVCAR-3 and SKOV-3 tumors at 24 h post injection, similar to the dose–response plateau observed in in vitro experiments, thus explaining the moderate therapeutic effect observed at the 40 mg/kg bi-weekly IP dose of D8P1C1. The imaging and xenograft data together indicate that, at this dose, the D8P1C1 monoclonal antibody specifically binds its target, ADAM17. Image-guided dose-escalation studies using an intraperitoneally administered tracer are necessary to further characterize the PET dose–response in these models. It is reasonable to consider potential clinical applications for ADAM17 immunoPET: noninvasive assessment of ADAM17 expression and activation in patients could identify likely responders and help guide treatment with therapeutic monoclonal antibodies or antibody-based agents.

When using murine models to explore the pharmacokinetics of a human IgG1 drug candidate, such as D8P1C1, there are inherent limitations. Mouse FcRn binds to human Fc with somewhat higher affinity but still engages in FcRn-mediated recycling. Thus, our model can recapitulate some of the Fc-dependent pharmacology but may overpredict the human biological clearance half-life [54]. Fortunately, in the NSG model, anti-D8P1C1 neutralizing antibodies are not likely to confound the interpretation of pharmacokinetics or influence hepatic or splenic trafficking of the radiotracer. D8P1C1 immunoPET in this model can, however, inform qualitatively on relative organ distribution in humans, estimate tumor targeting and de-risk later development. Another limitation of this study is that the human cancer xenograft model in immunocompromised mice does not fully explore the anti-tumor effect of a therapeutic antibody that can engage antibody-dependent cell cytotoxicity (ADCC) or phagocytosis (ADCP). Furthermore, drug pharmacokinetics can depend on tumor dynamics and engagement of immune effectors not captured in the NSG model. Future exploration of ^89^Zr-immunoPET-guided D8P1C1 therapy in murine syngeneic models of ADAM17-positive cancer is warranted.

Overall, our results reveal that D8P1C1 displays more promise in breast cancer in vivo models (78% tumor growth inhibition [15]), as compared to ovarian cancer models (25–45% tumor growth inhibition). The results are coherent with the fact that all four members of the EGFR family are known to be overexpressed in breast cancer [2], and, in contrast to ovarian cancer, ADAM17-dependent EGFR signaling is the major pathway driving breast cancer progression [2,5]. For decades, breast cancer treatment relied on surgery, radiation therapy, chemotherapy, or hormonal therapy [55]. Within the past 20 years, targeted antibody or small-molecule therapy has been sought as an effective measure to stymie the progression of breast cancer [56]. While progress has been made in targeting HER-2 in breast cancer, targeting EGFR has had limited success to date. As a first-line treatment for metastatic breast cancer, trastuzumab showed a response rate of 25% in HER2-positive patients [57]. However, EGFR antagonist development calls for further translational and clinical research to address unwarranted side effects. For example, the anti-EGFR mAb cetuximab, in combination with paclitaxel, resulted in dermatological toxicities during a phase 1 trial and was consequently abandoned [58]. A phase II randomized trial with cetuximab and carboplatin in women with metastatic TNBC showed limited efficacy [59].

Despite the modest therapeutic effect of mAb in ovarian cancer cell models, given that cell surface overexpression and upregulation of ADAM17 have been reported in a wide array of cancers [5], we modified the D8P1C1 mAb to generate a more potent antibody-based therapeutic, namely a bispecific T-cell engager antibody (BiTE) [16]. BiTEs have proven to be effective in targeting and killing cancer cells by redirected lysis [16,60]. This is achieved by simultaneously binding to a tumor-specific antigen and the CD3 subunit on the T cells, resulting in the formation of a cytolytic synapse [16]. As a sequel, the activated T cells release cytotoxic molecules and destroy the targeted cancer cell [16]. This improves the efficacy of cancer immunotherapies by enhancing T cell infiltration into tumor tissues. In preclinical models of CRC harboring KRAS (v-Ki-ras2 Kirsten rat sarcoma viral oncogene homologue) and BRAF (v-raf murine sarcoma viral oncogene homologue B1) mutations, BiTEs derived from cetuximab and panitumumab, both targeting EGFR, proved to be effective [47]. Cytotoxicity of an ADAM17-specific BITE, A300E, that recognizes the membrane-proximal cysteine-rich domain of human ADAM17 was previously shown in human prostate cancer cells in vitro, in the presence of T cells [61]. Since D8P1C1 [15] specifically targets tumor cells that depend on EGFR signaling, we evaluated the efficacy and specificity in vitro of the ADAM17-specific D8P1C1-derived BiTE using breast and ovarian cancer cell lines. The characterization of the D8P1C1-BiTE, though limited to in vitro studies, showed that the BiTE, in the presence of PBMC, is therapeutically more potent than the parental antibody, consistent with the general functional properties of BiTEs. In the future, we plan to evaluate the in vivo anti-tumor potency and tumor targeting of the D8P1C1 BiTE using mouse xenograft models, including patient-derived xenografts (PDX) [62]. The D8P1C1 mAb preferentially targets tumors and shows no discernible toxicity in mice models, and our current data related to PBMC (T cell) activation suggest a favorable, targeted cytokine release profile. Presumably, the administration of the D8P1C1 BiTE in vivo will not lead to unforeseen immune responses or off-target effects. However, further studies are needed with a broader panel of inflammatory mediators (e.g., IL-6, IL-10, and IFN) in humanized mouse models to understand its safety profile [16].

## 4. Materials and Methods

### 4.1. Cell Lines

The triple-negative breast cancer (TNBC) cell line MDA-MB-231 was grown in Dulbecco’s Modified Eagle Medium (DMEM), 10% Fetal Bovine Serum (FBS), 1% Penicillin/Streptomycin (P/S) and 2 mM L-Glutamine. The HER2-positive SKBR-3 cell line was cultured in McCoy’s 5a, 10% FBS and 1% P/S. The high-grade serous ovarian (HGSOC) cell line OVCAR-3 was grown in RPMI-1640, 10% FBS, 1% P/S, 10 mM HEPES and 0.2 units/mL insulin, while the Caov-3 (HGSOC) and SKOV-3 (non-HGSOC) cell lines were maintained in DMEM, 10% FBS and 1% P/S. HCC-827 (lung adenocarcinoma) and COLO-205 (colon cancer line) were cultured in RPMI-1640, 10% FBS and 1% P/S. These cell lines were purchased from American Type Culture Collection (ATCC, Manassas, VA, USA), subcultured (adhering to the instruction manual), and regularly checked for mycoplasma contamination.

### 4.2. Sandwich ELISA to Quantitate Endogenous Levels of Total EGFR and EGFR-P (Phosphorylated EGFR) in Lysates of MDA-MB-231, HCC-827, OVCAR-3 and SKOV-3 Cells, Untreated and Treated with D8P1C1

The generation and affinity-maturation of the anti-ADAM17 mAb have been described before [15]. The cancer cells were harvested in the log phase of growth, adjusted to 5 × 10^4^ cells/mL, and allowed to adhere and grow for 24 h in 24-well cell culture plates (Greiner Bio-One Cellstar). The cells were treated with 10 µg/mL of D8P1C1 and harvested after 4 h of treatment. Sandwich ELISA kits (Pathscan Cell Signaling Technologies) [31] were employed to quantitate total EGFR and EGFR-P in treated and untreated cells. The harvested cells were resuspended in 1X lysis buffer containing 1 mM PMSF, sonicated on ice and centrifuged for 10 min (18,800× *g*) at 4 °C. The supernatants (cell lysates) were collected for further study. Next, 100 µL of lysates (diluted to 1 mg/mL) was added to microwells previously coated with a mouse anti-EGFR mAb to capture both phosphorylated and non-phosphorylated EGFR. An anti-EGFR mAb raised in rabbit was used to detect the bound EGFR, while EGFR-P phosphorylated at Tyr1068 was detected by rabbit anti-phospho-EGF-receptor (Tyr1068) mAb. HRP-linked anti-rabbit antibody was used to recognize the bound antibody (detection antibody). Color was developed using the TMB substrate, and the data was recorded at 450 nm. Lysates from untreated cells served as controls. Comparison of EGFR and EGFR-P levels between treated and untreated groups was performed using the independent *t* test.

### 4.3. Sandwich ELISA to Quantitate the Cleavage of EGFR Ligands and Other ADAM17 Substrates from Cancer Cell Lines Untreated and Treated with D8P1C1

The cancer lines (MDA-MB-231, HCC-827, OVCAR-3, Caov-3, and COLO-205) were harvested in the log phase of growth and allowed to adhere to 24-well cell culture plates (Greiner Bio-One Cellstar, Monroe, NC, USA). The cells were treated with 20 µg/mL D8P1C1 or 1 µM small-molecule inhibitors of ADAM10 (GI254023X) [30], ADAM17 (TAPI-1) [29] or Matrix MetalloProteinases (Batimastat) [63] for 24 h. The supernatants of treated and untreated cells were harvested for sandwich ELISA (Invitrogen, Waltham, MA, USA) [31]. The cleaved EGFR ligands (ADAM17 substrates) released in the supernatants were bound to wells coated with corresponding capture antibodies. Biotinylated detection antibodies to EGFR ligands or ADAM17 substrates were added, followed by Streptavidin HRP. Color was developed using TMB. In COLO-205 colon cancer cells, the effect of D8P1C1 on the release of NICD1 was measured using the PathScan^®^ cleaved Notch1 (Val1744) sandwich ELISA Kit (Cell Signaling Technologies, Danvers, MA, USA) [64]. The data was recorded at 450 nm.

### 4.4. In Vivo Anti-Tumor Efficacy Studies

OVCAR-3 cells, grown in a monolayer culture, were harvested by trypsinization and implanted subcutaneously into the right flank of 6- to 8-week-old NSG mice (n = 5). Approximately 10 million cells (OVCAR-3) were injected per mouse. When tumors reached 100 to 150 mm^3^, the anti-ADAM17 mAb D8P1C1, prepared by diluting with sterile PBS, was injected intraperitoneally twice a week for four weeks at a dose of 40 mg/kg, with sterile PBS as a control. Tumor volume was calculated by the modified ellipsoidal formula: V = ½ (Length × Width^2^) [24]. Anti-tumor efficacy was calculated as (1-dT/dC) × 100, where dT is the final tumor volume minus the starting tumor volume from the treatment group, and dC is the final tumor volume minus the starting tumor volume of the control group. Error bars were calculated as standard errors of the mean (SEM). The experiments were executed in strict accordance with the Association for Assessment and Accreditation of Laboratory Animal Care and MSKCC Institutional Animal Care and Use Committee guidelines, and mouse body weight and health were monitored on a daily basis.

### 4.5. Radiochemistry, PET Imaging and Biodistribution

These studies were performed similar to another anti-ADAM17 mAb C12 [34]. The D8P1C1 mAb was buffer exchanged into HEPES buffer (pH 8.5, 0.5M) and conjugated with deferoxamine (DFO) using 5 molar equivalents of *p*-SCN-DFO (10 mM in DMSO, B-705, Macrocyclics Inc., Plano, TX, USA). The conjugation reaction was incubated at 37 °C for 90 min, and then purified by PD10 Sephadex G25 gel filtration chromatography (Cytiva Life Sciences, Marlborough, MA, USA) in 0.5M HEPES (pH 7.5) to yield DFO-D8P1C1 at a concentration of 1–2mg/mL. For radiolabeling, 100 MBq (~2.7 mCi) of ^89^Zr-oxalate in 1 M oxalic acid (5 µL, 3D Imaging LLC, Little Rock, AR, USA) was neutralized to pH 7 using 5 µL of 1 M K_2_CO_3._ The mAb DFO-D8P1C1 (~1.7mg, 1 mL) was added, gently mixed and incubated at 37 °C for 90 min. Radiolabeling progress, radiochemical purity and stability in PBS and mouse plasma at 37 °C were assessed by radio-instant thin layer chromatography (iTLC-SG, Agilent Technologies, Inc., Santa Clara, CA, USA) and read using an AR2000 scanner (Bioscan, Inc., Washington, DC, USA) [65]. ^89^Zr-DFO-D8P1C1 was purified by PD10 gel filtration chromatography, diluted to a final radioactive concentration (~0.3 mg/mL, 20 MBq/mL) with normal sterile saline and sterile filtered prior to in vivo administration. The mAb was also analyzed using size-exclusion HPLC with radio detection using an analytical TSKgel UP-SW3000 15 cm column (Tosoh Bioscience, King of Prussia, PA, USA). OVCAR-3 or SKOV-3 xenografts were established in NSG mice by inoculating with 10 × 10^6^ OVCAR-3 or 5 × 10^6^ SKOV-3 cells on the right flank of the mice (n = 3). Nine days after inoculation, ^89^Zr-DFO-D8P1C1 (50 µg (2.5 mg/kg), 3 MBq (80 µCi) in 150 µL) was administered intravenously by tail vein. ^89^Zr activity of radiochemical samples and syringe doses were measured using a CRC-55tR dose calibrator (Capintec Inc., Florham Park, NJ, USA). MicroPET/CT imaging was performed on an Inveon (Siemens Medical Solutions USA, Malvern, PA, USA) at 24 h, 72 h, and 168 h. PET/CT visualization, segmentation, and region-of-interest (ROI) analysis was performed using Amide 1.0.6 [66] and represented as percent of injected dose per cc (assumed 1 cc = 1 g) of tissue (%ID/g). For ex vivo biodistribution, tissues were dissected and weighed, with radioactivity concentrations determined by measurement in a γ-well counter (Hidex AMG, Turku, Finland), represented in terms of %ID/g, and plotted using Prism10 (GraphPad Software, Boston, MA, USA).

### 4.6. Generation of D8P1C1-BiTE by Fusing the Single Chain Variable Fragment (scFv) of D8P1C1 to the scFv of an Anti-CD3 mAb

The single chain variable regions of D8P1C1 were fused to those of an anti-CD3 mAb specific for the human CD3 epsilon chain [37] in the following order: VLD8P1C1-linker(GGGGSGGGGSGGGGSSG)-VHD8P1C1-linker(GGGGS)-VHanti-CD3-linker(GGSGGSGGSGGSGG)-VL-anti-CD3 [16]. A removable Fc-tag was then fused to the C-terminus of this construct. The fused gene construct was cloned, expressed, and purified from HEK293 cells using a custom-made pcDNA^TM^ 3.1^+^ vector [13,15]. The C-terminal Fc-tag was used to facilitate protein-A affinity chromatography and was removed afterwards by thrombin cleavage. The final purification was performed on a SD-200 column (size-exclusion chromatography, SEC). The purified BiTE elutes around 50 kDa (co-migrates with ovalbumin) on the SD-200 column and migrates at 50 kDa on SDS-PAGE (under reducing as well as non-reducing conditions). Peripheral blood mononuclear cells (PBMC) were purchased from ATCC (one-time use), and Alamar cell viability assays were performed in the presence and absence of PBMC using breast and ovarian cancer cell lines. Before the addition of the Alamar dye, the medium was aspirated, and attached cells were washed gently once with PBS, at pH 7.4. The dye (10% *v*/*v*) was added to appropriate media, and absorbances were recorded as described before [13,15,44,45]. Briefly, cell viability was measured spectrophotometrically by absorbance at 570 and 600 nm. Cell viability was calculated using the following formula:

Percentage difference between treated and control cells = ((O2 × A1) − (O1 × A2) × 100)/((O2 × P1) − (O1 × P2))

O1 = molar extinction coefficient (E) of oxidized alamarBlue^®^ (Blue) at 570 nm, O2 = E of oxidized alamarBlue^®^ at 600 nm, A1 = absorbance of test wells at 570 nm, A2 = absorbance of test wells at 600 nm, P1 = absorbance of growth control well (cells plus alamarBlue^®^ but no test agent) at 570 nm, and P2 = absorbance of growth control well (cells plus alamarBlue^®^ but no test agent) at 600 nm.

### 4.7. Statistical Analysis

The data from all in vitro and cell-based assays are representative of triplicate determinations. Statistical analysis was performed using IBM SPSS version 29. *p*-values were calculated using either one-way ANOVA with a Dunnett’s multiple comparison post hoc test, or one- or two-tailed independent *t* test analysis, as indicated on figures. A *p*-value of <0.05 was considered significant.

## Figures and Tables

**Table 1:** Longitudinal, image-based biodistribution by ROI (region of interest) analysis of ^89^Zr-DFO-D1P1C1 PET/CT imaging in xenograft tumor-bearing mice (n = 3).

Cell Line		OVCAR-3			SKOV-3	
Time (Hours)	24	72	168	24	72	168
Tumor	12.08 ± 0.80	10.74 ± 1.03	7.89 ± 0.54	6.43 ± 4.54	6.85 ± 4.41	0.45 ± 0.33
Heart	7.24 ± 1.99	2.20 ± 0.66	2.51 ± 0.26	4.52 ± 2.01	2.52 ± 1.17	0.60 ± 0.19
Lung	5.39 ± 0.61	3.67 ± 0.34	2.56 ± 0.52	3.44 ± 1.04	2.74 ± 0.88	1.18 ± 0.65
Liver	21.25 ± 0.45	16.25 ± 2.00	12.88 ± 0.92	12.22 ± 3.01	12.04 ± 4.65	2.89 ± 1.20
Intestine (S + L)	5.00 ± 0.81	2.51 ± 1.11	0.61 ± 0.21	3.12 ± 0.81	2.37 ± 0.26	0.20 ± 0.02
Kidney	7.27 ± 2.25	4.93 ± 2.59	3.51 ± 0.66	5.46 ± 2.28	2.77 ± 0.56	0.50 ± 0.20
Muscle	1.43 ± 0.37	0.68 ± 0.38	0.48 ± 0.03	0.82 ± 0.39	0.77 ± 0.25	0.26 ± 0.38
Bone/Tibia	9.61 ± 1.01	9.91 ± 1.17	7.11 ± 2.07	2.53 ± 0.61	2.80 ± 1.62	0.36 ± 0.04

## Data Availability

The original contributions presented in this study are included in the article/Supplementary Material. Further inquiries can be directed to the corresponding authors.

## Supplementary Materials

The following supporting information can be downloaded at: https://www.mdpi.com/article/10.3390/ijms27072936/s1. References [67,68] are cited in the Supplementary Materials.

## Abbreviations

ADAM: A Disintegrin And Metalloproteinase; AREG: Amphiregulin; BiTE: Bispecific T-cell Engager Antibody; CXCL: C-X-C Motif Chemokine Ligand; DFO: Desferrioxamine; EGF: Epidermal Growth Factor; EGFR: Epidermal Growth Factor Receptor; HGSOC: High-grade Serous Ovarian Cancer; mAb: Monoclonal Antibody; NSG: Nude SCID Gamma; PET: Positron Emission Tomography; PBMC: Peripheral Blood Mononuclear Cells; PARP: Poly ADP Ribose Polymerase Inhibitors; ROI: Region of Interest; TAPI-1: Tumor Necrosis Factor-α (TNF-α) Protease Inhibitor-1; TNBC: Triple-Negative Breast Cancer; TGFα: Transforming Growth Factor-alpha.
