# Supplementary material for: Characterization of the D8P1C1 Anti-ADAM17 Inhibitory Monoclonal Antibody and Generation of Its Bispecific T-Cell Engager Derivative
Source: Int J Mol Sci. 2026 Mar 24;27(7):2936. doi: 10.3390/ijms27072936 (PMC13073511; doi:10.3390/ijms27072936)
Supplement: Supplementary file 1 [file ijms-27-02936-s001.zip › ijms-4169324-supplementary.pdf]

Supplementary Materials

A B

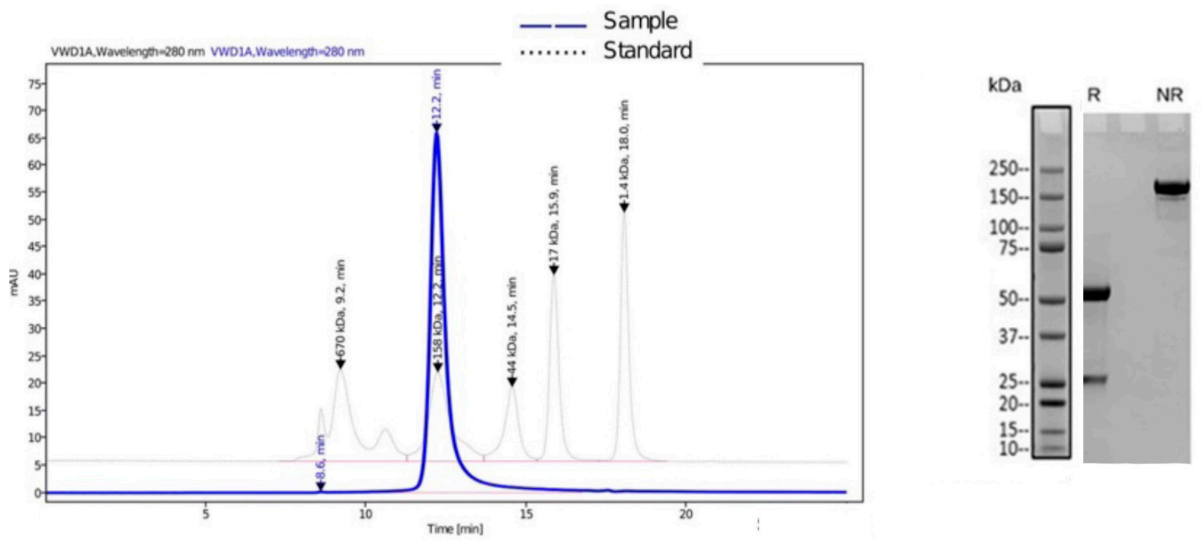

Signal: VWD1A,Wavelength=280 nm

RT [min]	Height	Width [min]	Area	Area%
8.6	0.2	0.5	2.8	0
12.2	66.0	7.9	2412.8	>99
		Sum	2415.55	

Figure S1. Large scale production of the D8P1C1 mAb. The D8P1C1 mAb was produced in Expi293 and purified to 99% homogeneity. The final endotoxin limit was <0.1 EU/mg to facilitate animal studies. The mAb migrates with a native molecular weight of 150 kDa (non-reducing conditions, NR) as indicated by SEC HPLC, with the heavy and light chains migrating at 50 and 25 kDa respectively on SDS-PAGE under reducing (R) conditions.

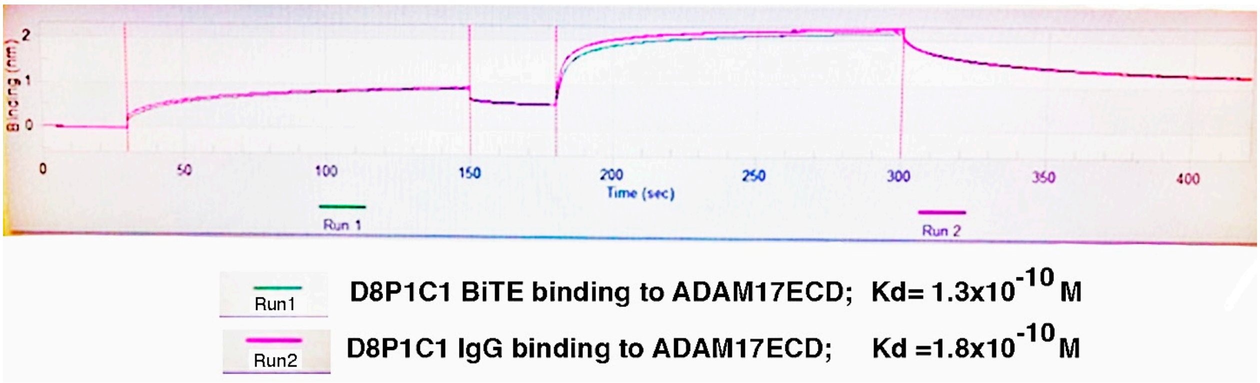

Figure S2. Biolayer interferometry to quantitate the binding of D8P1C1 (IgG or BiTE) to ADAM17 ECD. Protein A biosensors were loaded with 50 µg/ml solution of purified IgG or BiTE-Fc-tag, washed with HBS (20 mM HEPES, 150mM NaCl pH7.4), followed by addition of purified ADAM17 ECD. Affinities (Kd) were calculated using the BLItzPro software [67,68]. The ADAM17 ECD showed no binding to protein A Sepharose or to an unrelated anti-ADAM10 IgG 1H5 [13]. The y-axis represents the change in binding thickness (nm shift), the x-axis tracks how the binding changes over time.

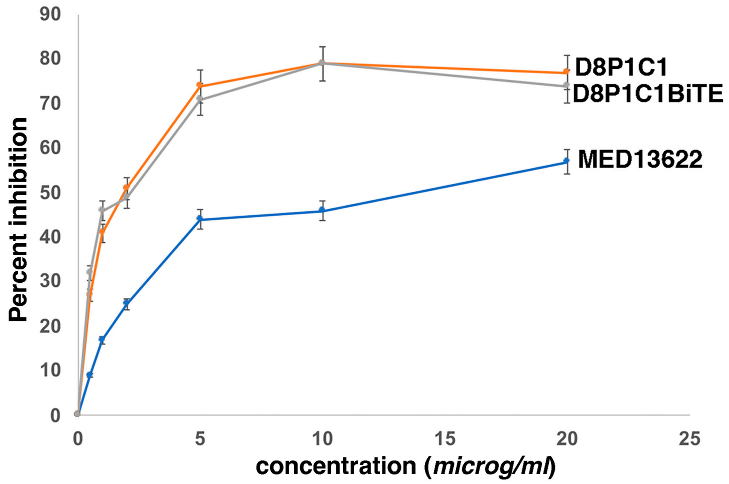

Figure S3. Alamar blue cell viability assay with the TNBC line MDA-MB-231. The effect of anti-ADAM17 mAbs and D8P1C1 BiTE in absence of PBMC. The anti-ADAM17 mAb MED13622 [19] was included in the assay for comparison (n=3). The IC50 values for the two IgG's and the BiTE, measured using the MDA-MB-231 cell line, were: IC50 of D8P1C1 IgG: 5 nM; IC50 of D8P1C1 BiTE: 12 nM; IC50 of MED13622 anti-ADAM17 mAb: 23 nM.

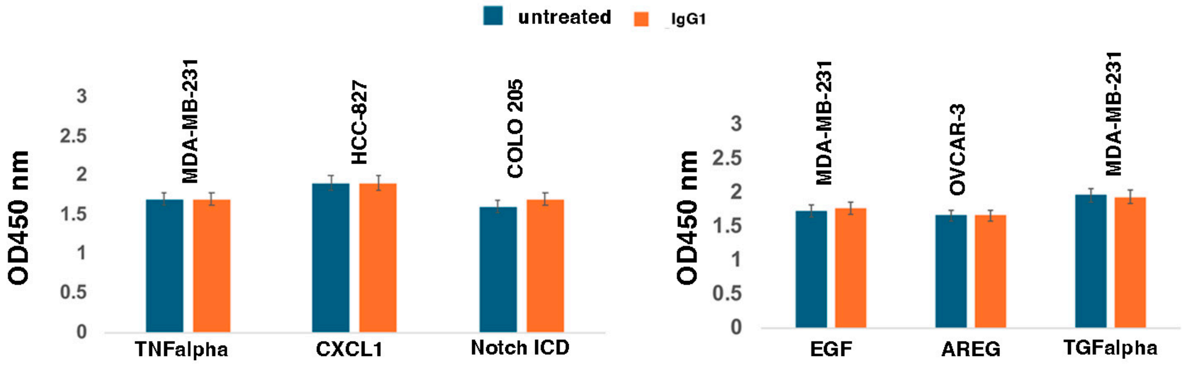

Figure S4. The isotype control human IgG1 (20 μg/ml) does not inhibit the shedding of ADAM17 substrates (left panel) or EGFR ligands (right panel) from the cell surface of cancer cells. Sandwich ELISA was employed to quantitate the shedding (or cleavage) of ADAM17 substrates and EGFR ligands. For Notch, we measured the release of the Notch intracellular domain or NICD1 in the cell lysates. In all cases, the IgG1 control was undistinguishable from the untreated cells (n=3).

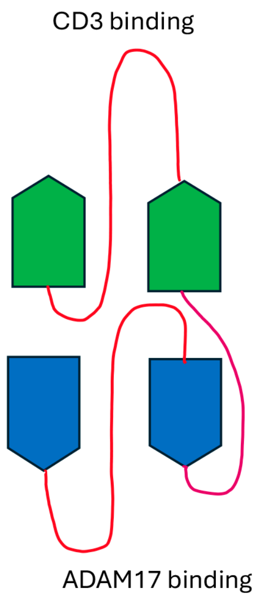

Figure S5. Schematic illustration of the D8P1C1 BiTE.

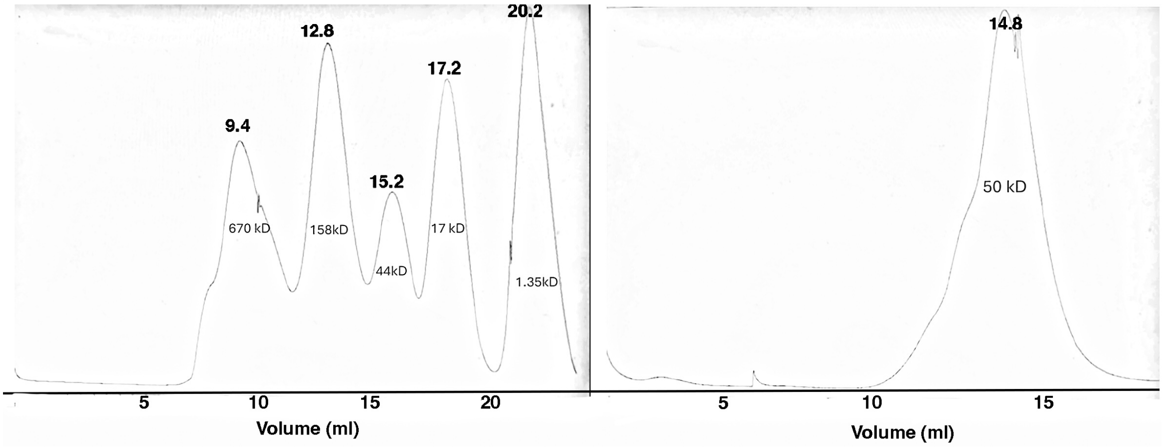

Figure S6. SD-200 elution profile of the D8P1C1 BiTE. The numbers in black represent elution volumes in ml. Left panel: elution profile of protein standards with defined molecular weights. Right panel: elution profile of the D8P1C1 BiTE.

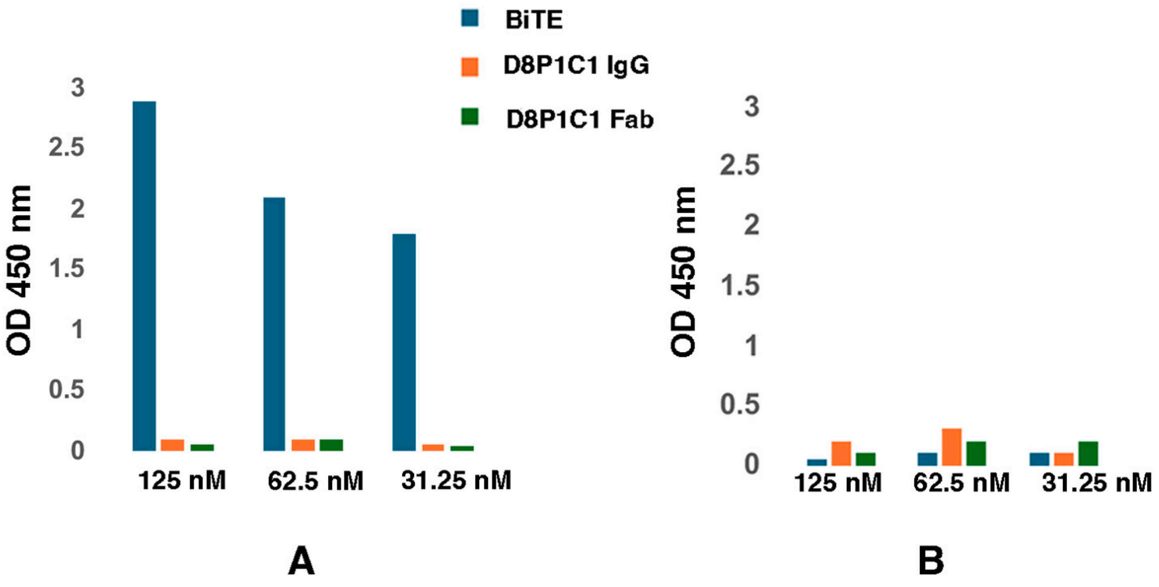

Figure S7. Binding of BiTE (Fc-tagged version), D8P1C1 IgG, and D8P1C1 Fab, to immobilized PBMC (A) and HL-60 cells (B) purchased from ATCC. The Fc-tagged version of the BiTE, just after protein A Sepharose elution, was used for the ELISA assay. The bound Fc-tagged BiTE was detected by goat anti-human Fc secondary mAb conjugated to HRP (Invitrogen). The bound whole IgG was detected using the same secondary mAb conjugated to HRP. The bound Fab was detected by goat anti-Human IgG (H+L), Jackson Immunoresearch.
